# Effect of pentagonal-coordinated surface on crystal nucleation of an undercooled melt

**DOI:** 10.1038/s41598-018-32594-w

**Published:** 2018-09-25

**Authors:** A. Pasturel, N. Jakse

**Affiliations:** Univ. Grenoble Alpes, CNRS, Grenoble INP, SIMaP, F-38000 Grenoble, France

## Abstract

Bringing a liquid into contact with a solid is known to generally promote crystal nucleation at the freezing temperature. In contrast, it is much more difficult to conceive that a solid surface may hinder nucleation and favor large undercooling effects. Here we report on *ab initio* and classical molecular dynamic simulations to capture the underlying structural mechanism responsible for this striking effect. We find that the substrate/liquid interactions exert an important influence on in-plane ordering of the adjacent liquid layers in the undercooling regime. In particular, we identify that the presence of atomic arrangements with five-fold symmetry (FFS) on the substrate surface in the form of pentagonal atomic motifs allows the liquid to be undercooled well below its freezing temperature. Our findings clearly demonstrate that this pentagonal-coordinated surface enhances the presence of local arrangements with FFS in the adjacent liquid layers that prevents the crystal nucleation. Finally we suggest new technological developments to attain large undercooling effects.

## Introduction

Undercooling, a state where liquids do not solidify even below their normal freezing temperatures, can only be achieved in liquids that do not contain seeds that may trigger crystallization. Over the last 60 years, the existence of deep undercooling in metallic liquids^1^ led to suggest that the homogeneous nucleation mechanism responsible for formation of the crystal phase must present a large barrier to phase change^2^. To explain this large barrier, Frank^3^ proposed that the local structures in undercooled metallic liquids displayed a significant degree of five-fold symmetry (FFS), incompatible with the translational symmetry of the crystal. The evidence for such local structures in undercooled metallic liquids came out only recently using *ab initio* and classical molecular dynamics simulations^4–6^ and when containerless-based processing methods were combined with scattering techniques^7,8^. Indeed, the application of containerless-based methods prevents the container-wall heterogeneous nucleation.

Yet, Schulli *et al*.^9^ recently demonstrated that a solid surface may favor remarkable undercooling by acting as a template for a liquid. Using an X-ray scattering technique, they report a very low value of the freezing point of the liquid Au-Si eutectic alloy, about 120 K below the eutectic temperature, *T*_E_ = 636 ± 5 K, when this liquid is in contact with a specially decorated silicon surface, the Si (111)-(6 × 6) Au reconstruction^10^. Moreover, they found that the degree of undercooling is significantly reduced when the same liquid is exposed to four-fold and three-fold coordinated surfaces, *i.e*. Si (001) and Si (111) surfaces. Interestingly, the (6 × 6) reconstruction features pentagonal Au arrangements (see Fig. 4 of ref.^10^) which are also typical of local atomic arrangements present in the eutectic liquid^11^. Then, authors proposed that the (6 × 6) reconstruction offers sites to stabilize these local structures in the eutectic liquid well below the eutectic temperature, giving rise to in-plane ordering which impedes the crystallization of the liquid. Also of relevance to this kind of in-plane ordering is the experimental work by Reichert *et al*.^12^ who found pentagonal arrangements of lead atoms in liquid Pb close to a (001) surface of Si. Nevertheless, when the eutectic alloys freezes on the (6 × 6) reconstruction, the resulting gold crystals form in random orientations. Such a result suggests that the (6 × 6) reconstruction has no orienting role in solidification and the mechanism of crystal nucleation remains to be explored.

Solid/liquid systems that present interface-induced atomic layering, *i.e*. out-of-plane ordering like Al liquid on Al_2_O_3_^13^, also show significant undercooling. However, the in-plane structure of these interfaces is not resolved when it is expected to be more crucial than the perpendicular one in controlling the stability of a liquid against crystallization^14^. Therefore, despite its importance, experimental characterization of the in-plane ordering is still at its beginning and in this context, atomistic simulations can play a vital role to provide a comprehensive view of the nature of the ordering within the liquid layers adjacent to substrates^15,16^ and its relation to freezing or its inhibition.

In this work, to further address the important question of the in-plane ordering in connection to the undercooling ability, we have performed a series of *ab initio*^17^ and classical molecular dynamics (MD)^18–20^ simulations of the liquid Au-Si eutectic alloy in contact with two different silicon substrate surfaces, namely Si(001) and the Si (111)-(6 × 6) Au reconstruction. By comparing the two systems, we find that the substrate/liquid interactions affect in-plane ordering of the adjacent liquid layers dramatically. In particular, we observe that the in-plane ordering induced by Si (001) becomes quite different from the short-range order with a significant degree of FFS found in the bulk liquid upon undercooling. In contrast, we evidence that the Si (111)-(6 × 6) Au reconstruction favors this short-range order well below the eutectic temperature. We demonstrate that this finding can be related to the high degree of FFS displayed by this reconstruction. Furthermore, we show that the conservation of the liquid state induced by this interface in the undercooling regime hinders the formation of gold crystals.

## Results

### In-plane ordering in the liquid regime

**Figure 1:**
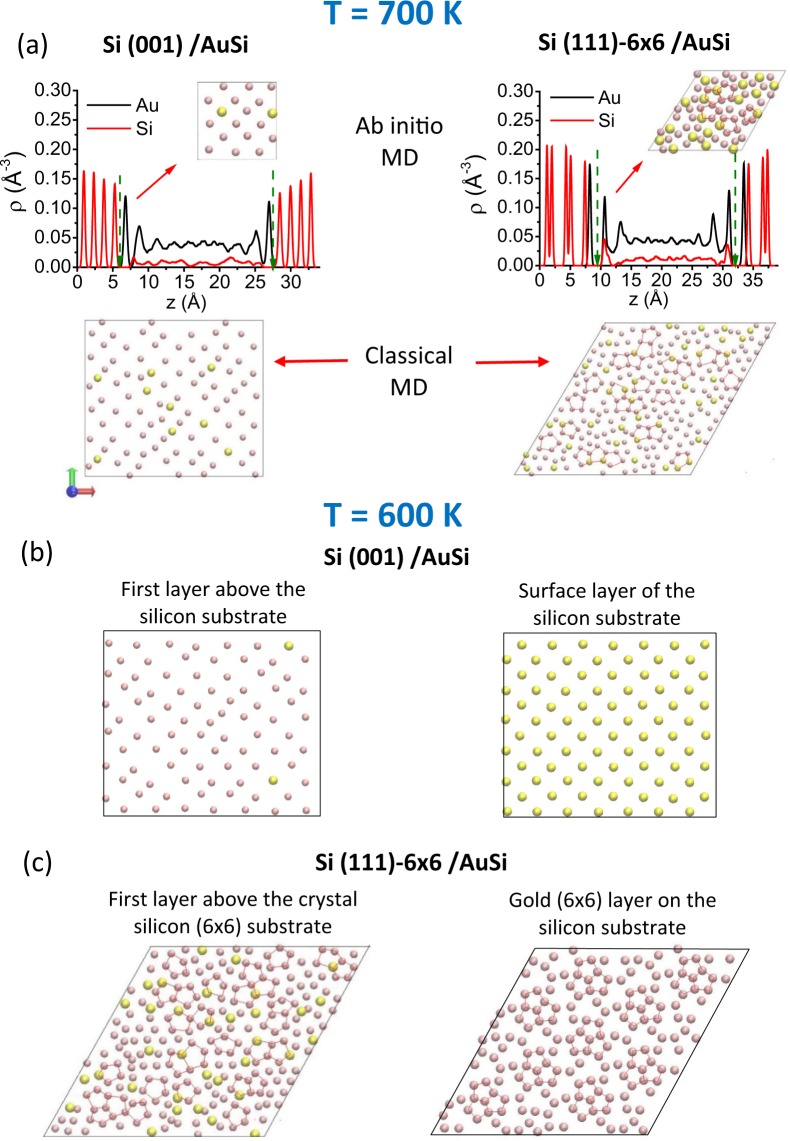
(**a**) Density profile along the *z* direction and first layer above the Si substrate at *T* = 700 K for the Si (001)/AuSi interface (left part), and Si (111)-(6 × 6)/AuSi interface (right part), from AIMD simulations (upper panels). First layer above the Si substrate from large scale classical MD simulations with the MEAM potential (lower panels). The green dotted line arrows give the position of both interfaces in each simulation box. For *T* = 600 K, the first layer above the Si substrate from large scale classical MD simulations for (**b**) Si (001)/AuSi interface, and (**c**) Si (111)-(6 × 6)/AuSi interface where pentagonal atomic configurations are highlighted with red lines (left panel). The respective right panels show the surface layer of Si (001) substrate and the (6 × 6) gold layer on the Si (111) surface. For the Si (111)-(6 × 6)/AuSi interface, pentagonal atomic configurations are highlighted with red lines. For all simulation boxes, the axis orientations are given by the red (*x*-axis), green (y-axis) and blue (z-axis) thick arrows. Si atoms are in yellow color, and Au atoms are in pink color.

From *ab initio* molecular dynamics (AIMD) calculations, we first quantify the structural properties of the interfacial liquid. Upper panels of Fig. 1(a) display the atomic density profiles for Si and Au, which are averaged in planes parallel to the two interfaces, obtained by AIMD simulations at *T* = 700 K, 64 K above *T*_E_. We emphasize that the strong self-regulation mechanism at the interfacial regions enables the liquid to rapidly reach equilibrium at 700 K and thus the simulation time is sufficient. The density profile in the liquid region clearly shows that a few liquid layers are formed near each interface. The layering effect decays rapidly with depth from each interface and examination of the two interface profiles do not reveal any significant differences. To extend this structural description, Fig. 1(a) shows also snapshots of the first liquid layer at *T* = 700 K for Si (001)/Au-Si and (6 × 6)/Au-Si interfaces. The difference between the two interfaces is now striking. We find a clear in-plane ordering for the liquid layer in contact with the Si (001) surface while an apparently disordered in-plane atomic structure is observed in the adjacent layer to the (6 × 6) reconstruction.

To confirm this result, we performed classical MD with longer simulation times and larger computational supercells shown in Fig. S3 and described in details in (See Supplementary Information). Lower panels of Fig. 1(a) clearly indicate that in-plane ordering in the first liquid layer is different in the two substrate orientations, in agreement with *ab initio* based calculations. At the (001) interface, we observe that the chemical composition of the first liquid layer is smaller than that of the eutectic alloy, namely 10 at% versus 19 at% of Si. Moreover, both Au and Si atoms display mainly a fourfold coordination, similar to that of the Si (001) surface. However, Fig. S4 (See Supplementary Information) shows that the subsequent liquid layers rapidly lose this local ordering and display pentagonal Au-based arrangements which gradually increase from the interface to recover a short-range order with a degree of FFS similar to that of the bulk-like AuSi liquid, namely 25% of FFS^11^. Interestingly, we do not find any change in the chemical composition of the first liquid layer at the (6 × 6) interface and we observe significant pentagonal Au-based arrangements. To quantify them, we performed a common-neighbor analysis^21^. In this method, pairs of atoms are classified by four Honeycutt-Andersen indices that are determined by the number of nearest neighbors and their connectivity. For instance, the 1551 pair in Fig. S3 represents the motif with FFS, which allows describing both local ordering in the liquid phase and the pentagonal structure in the (6 × 6) reconstruction. Our common-neighbor analysis reveals that the percentage of pentagons in the first liquid layer is greater than that in the bulk liquid, namely 50% versus 25%^11^. Moreover, the degree of FFS in the first liquid layer becomes close to that of the (6 × 6) reconstruction, *i.e*. 65%^9^. Our findings indicate that the (6 × 6) surface seems to favor FFS in the adjacent liquid layer at *T* = 700 K.

### In-plane ordering in the undercooled regime

To go further, we inspect how the in-plane ordering in the liquid layers changes upon undercooling and what is its degree of correlation with the structure of the underlying surface. Fig. 1(b,c) show the main characteristics of the interface structure at *T* = 600 K, 36 K below *T*_E_, for Si (001)/Au-Si and (6 × 6)/Au-Si, respectively, including the first liquid layer as well as the topmost crystal layer. The left panel of Fig. 1(b) shows that the first liquid layer in contact with the (001) surface exhibits a significant change upon cooling, characterized by the almost total absence of Si atoms and Au atoms arranged mainly with the fourfold coordination similar to that of the Si (001) surface, revealing a pronounced in-plane ordering. As a matter of fact, comparison with the right panel in Fig. 1(b) shows that Au atoms in this layer are very close to the extrapolated lattice positions of a next substrate layer. Moreover, upon moving further from the (001) surface, the fourfold coordination becomes also visible as shown in Fig. S5 (See Supplementary Information), meaning a greater influence of the substrate on the liquid phase. In addition, one expects that this in-plane ordering will lead to a reduced mobility of Au atoms lying in it. This is indeed what is seen in the in-plane diffusion profiles in Fig. S8 (See Supplementary Information) where we report the self-diffusion coefficients in the liquid phase as a function of the distance from the interface as described in (See Supplementary Information). We obtain that the mobility of atoms slows down continuously when approaching the interface and drops abruptly as the ordered Au layer is reached. We also report that diffusivity calculated at *T* = 600 K in the bulk-like part of the interfacial liquid is the same than that obtained in the bulk liquid. We will discuss below how this pronounced in-plane ordering may influence nucleation.

**Figure 2:**
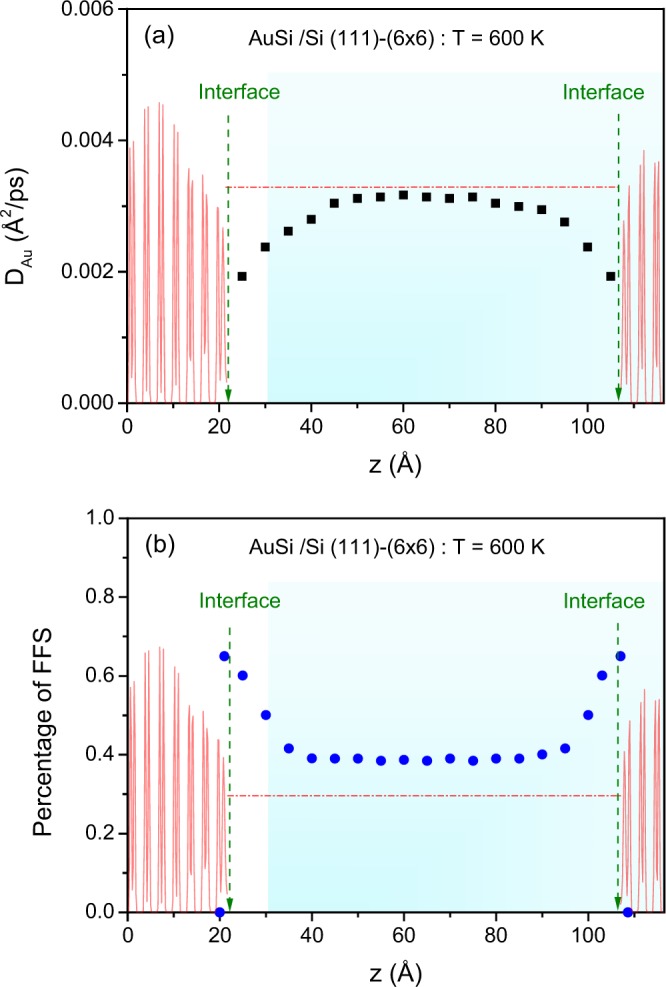
Results from the large scale classical MD simulations with the MEAM potential at *T* = 600 K for the Si (111)-(6 × 6)/AuSi interface determined in the liquid slab for (**a**) the self-diffusion coefficient for Au as a function of *z*, and (**b**) the percentage of five-fold symmetry as a function of *z*, The shadowed red lines in both panels represent the density profiles in arbitrary units of the crystalline substrate for visual purpose only, in order to highlight the AuSi liquid slab *z*-range. In each case, the green dotted line arrows mark the positions of both interfaces, and the red dot-dashed horizontal line marks the corresponding value of the bulk eutectic liquid^19,20^.

In contrast, the left panel of Fig. 1(c) shows that the chemical composition and local ordering of the first liquid layer in contact with the (6 × 6) surface do not exhibit any dramatic change upon cooling. As already observed at 700 K, the first liquid layer displays pentagonal Au-based arrangements. Furthermore, a close inspection indicates that the degree of FFS slightly increases as well as the connectivity between pentagonal arrangements. However, comparison with the (6 × 6) reconstruction displayed in the right panel of Fig. 1(c) shows that pentagonal arrangements in the first liquid layer are not captured by the Au pentagons of the reconstruction. In other terms, we do not detect any “epitaxial” relationship of Au pentagons of the first liquid layer with those of the (6 × 6) reconstruction. This interface-conditioned scenario is also confirmed by the fact that subsequent layers display a less pronounced degree of FFS and interconnectivity than in the first layer with the trend to recover a bulk-like behavior, as shown in Figs S6 and S7 (See Supplementary Information). However, some striking effects of the (6 × 6) reconstruction on the interfacial liquid can be seen in Fig. 2. The diffusion constant profile in Fig. 2(a) is significantly less abrupt than that corresponding to the (001) surface displayed in Fig S8 and is characterized by a value in the bulk-like part lower than that of the bulk eutectic liquid^19^, in accordance to a higher degree of FFS as shown in Fig. 2(b).

### Crystal nucleation

**Figure 3:**
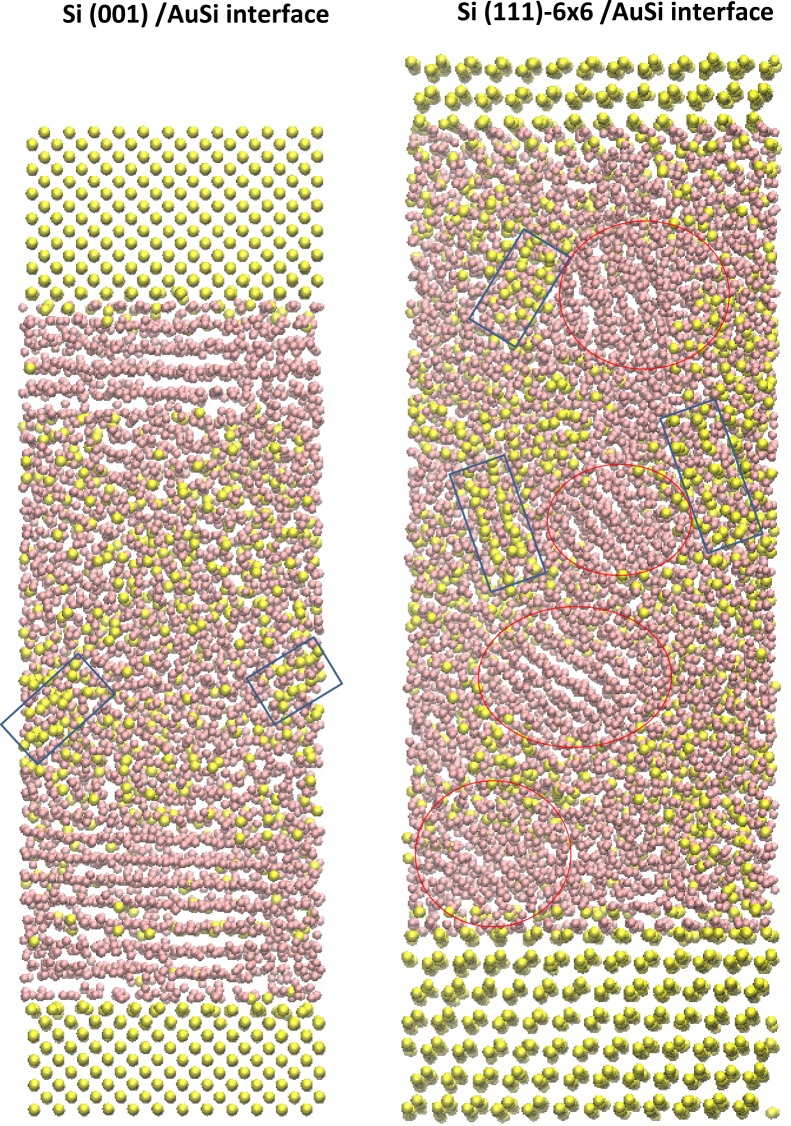
Snapshot of the simulation box for the classical MD with the MEAM potentials during the solidification process for Si (001)/AuSi interface at *T* = 570 K (left panel), and Si (111)-(6 × 6)/AuSi interface at *T* = 500 K (right panel). Si atoms are in yellow color, and Au atoms are in pink color. The red circles in the right panel highlight some of gold grains formed. Blue rectangles in both panels show grains of Au_3_Si metastable phase.

The large difference that exists in the in-plane structure of the adjacent liquid layer when comparing the Si (001) surface with the (6 × 6) reconstruction suggests also some differences in the mechanism of crystal nucleation. First, we can suspect that the in-plane gold ordering observed at the Si (001) interface and its development in few subsequent adjacent layers at *T* = 600 K may initiate the formation of gold crystals. Then, we quenched the Si (001)/Au-Si system at *T* = 570 K (at time t = 0) which corresponds to the experimental onset of freezing of the eutectic liquid in contact with the (001) surface. The system is then monitored for nucleation (for 0 < t < 30 ns) while keeping the temperature constant. Twenty independent simulations are used to determine a mean nucleation time, τ, with a good statistic. We find that nucleation always starts at τ = 5 ± 1 ns. Moreover, Fig. 3(a) indicates that it is mainly characterized by the formation of gold grains displaying a parallel in-plane epitaxial relationship with the surface. Such a result is in close agreement with experiments^22^ which show that the preferred orientation relationship between the solidified Au and the Si(001) surface is: [100]Au(001)||[100]Si(001) (see Fig. S9 for more details). We also detect another phase in the bulk part of the interfacial liquid due to the quench procedure as discussed below.

For the (6 × 6) reconstruction, we adopt a different strategy as this reconstruction is experimentally known to inhibit nucleation of the eutectic liquid^9^. Starting from 600 K, the system is cooled down to 500 K with 10 K intervals. To detect nucleation events, we performed fifty independent simulations. From 600 K to 520 K, we do not detect any nucleation events in the fifty runs within the simulation time of 30 ns. At 500 K, we find that the gold nucleation starts at τ = 15 ± 1 ns. Note that the experimental onset of freezing is 513 K for the (6 × 6) reconstruction. Interestingly, Fig. 3(b) shows that gold grains form in a random orientation. Moreover, we also observe other regions well organized at the atomic scale but with a silicon composition close to 25 at% Si. Note that this phase is also observed during the freezing of the eutectic alloy in contact with the (001) surface. This additional phase can be related to the formation of the metastable Au_3_Si compound obtained when the bulk eutectic liquid is rapidly quenched^23–25^.

Next to better quantify the role played by the reconstruction on the freezing of the eutectic liquid, we also quenched the bulk eutectic liquid using the same procedure and with two different computational cells, 1372 and 10976 atoms respectively. We find that the crystal nucleation starts at *T* = 590 K with τ = 12 ± 1 ns 15 ± 1 ns respectively in the 1372 and 10976 atoms computational cells. In Fig. S10 (See Supplementary Information) we show configurations obtained after nucleation at *T* = 590 K. Comparison with Fig. 3(b) suggests that they are similar to those observed in the (6 × 6)/Au-Si system at T = 500 K, characterized by the appearance of gold grains as well as Au_3_Si metastable phase. Then, we can consider a similar mechanism of nucleation but at a lower temperature in the interfacial liquid.

## Discussion

To further elucidate the microscopic mechanism which hinders the crystal nucleation in the interfacial liquid we propose the following scenario. First, the degree of FFS in the bulk-like part of the interfacial liquid is higher than the one found in the bulk liquid, (see Fig. 2(b)). Such an increase induced by the striking (6 × 6)-induced enhancement of FFS in the first adjacent layers favors undercooling effects since it gives very stable local structures incompatible with long-range periodicity. In addition, in Fig. S7 (See Supplementary Information), it is interesting to note that a majority of pentagonal arrangements in the bulk-like part of the interfacial liquid display a chemical composition similar to that of the eutectic alloy, namely Au_4_Si. This is in contrast to predominant pentagonal Au arrangements found in the undercooled bulk melt^11^. Thus, our findings provide a novel perspective for understanding how the crystal nucleation in a liquid may be inhibited by a surface. Yet the generality and limitation of this scenario need to be checked carefully in the future.

The situation we meet with the (6 × 6) reconstruction illustrates very well how a substrate surface may delay solidification by preserving the local ordering of the liquid phase, here pentagonal local structures. We are aware that AuSi is often considered as a liquid with unusual properties but the pentagonal local ordering is found in a vast range of liquids and on the basis of the results reported here, we clearly establish that solid-liquid interfaces that favor pentagonal local ordering should lead to deeper undercooling. This may have important technological implications as the use of containers with inner surfaces which mimic a liquid to obtain undercooling.

## Methods

We performed ab initio molecular dynamic (AIMD)^17^ within the same approximations used in the study of the bulk Au-Si liquid^10^. Moreover, we complemented AIMD simulations with classical MD simulations using the LAMMPS code^18^ using appropriate interatomic potentials^19,20^. The starting configurations for the solid/liquid interfaces were constructed to simulate the solid/liquid systems in the two crystallographic orientations, Si (001)/Au-Si, and (6 × 6)/Au-Si. All details are described in the (Supplementary Material See Supplementary Information). See Figs S1 and S2 in particular.

Structural and dynamic properties of the liquid phases in the two interfaces are determined in the investigated temperature range from the MD simulation trajectories produced in the canonical (NVT) ensemble. For *ab initio* calculations, each run was continued up to 110 ps while longer simulations (up to 30 ns) were performed to study nucleation occurrence using classical MD simulations. Note that these latter simulations are more than twenty times longer than the simulation times required to determine the stability limits of the bulk liquid phase, *i.e*. the liquid-solid phase boundaries^19^.

## Electronic supplementary material

Supplementary Information

## References

[1] Turnbull D (1952). Kinetics of Solidification of Supercooled Liquid Mercury Droplets. J. Chem. Phys..

[2] Kelton, K. F. Crystal Nucleation in Liquids and Glasses. in *Solid State Physics*, edited by Ehrenreich, H. & Turnbull, D. (Academic Press, Boston) **45**, 75–177 (1991).

[3] Frank FC (1952). Supercooling of Liquids. Proc. R. Soc. London A.

[4] Jakse N, Pasturel A (2003). Local order of liquid and supercooled zirconium by ab initio molecular dynamics. Phys. Rev. Lett..

[5] Kang J, Zhu J, Wei S-H, Schwegler E, Kim Y-H (2012). Persistent medium-Range Order and Anomalous Liquid Properties of Al_1−x_Cu_x_ alloys, Kang, J. *et al*. Phys. Rev Lett..

[6] Hu YC, Li FX, Li MZ, Bai HY, Wang WH (2015). Five-fold symmetry as indicator of dynamic arrest in metallic glass-forming liquids. Nature communications.

[7] Schenk T, Holland-Moritz D, Simonet V, Bellissent R, Herlach DM (2002). Icosahedral short-range order in deeply undercooled metallic melts. Phys. Rev. Lett..

[8] Kelton KF (2003). First X-ray scattering studies on electrostatically levitated metallic liquids: demonstrated influence of local icosahedral order on the nucleation barrier. Phys. Rev. Lett..

[9] Schülli TU (2010). Substrate-enhanced supercooling in AuSi eutectic droplets. Nature.

[10] Grozea D (1998). Direct methods determination of the Si (111)-(6×6) Au surface structure. Surf. Sci..

[11] Pasturel A, Tasci ES, Sluiter MH, Jakse N (2010). Structural and dynamic evolution in liquid Au-Si eutectic alloy by ab initio molecular dynamics. Phys. Rev. B.

[12] Reichert H (2000). Observation of five-fold local symmetry in liquid lead. Nature.

[13] Oh SH, Kauffmann Y, Scheu C, Kaplan WD, Rühle M (2005). Ordered liquid aluminum at the interface with sapphire. Science.

[14] Greer AL (2006). Liquid metals: Supercool order. Nature Mat..

[15] Kang J (2012). Atomically Abrupt Liquid-Oxide Interface Stabilized by Self-Regulated Interfacial Defects: The Case of Al/Al_2_O_3_ Interfaces. Phys. Rev. Lett..

[16] Guerdane M, Teichler H, Nestler B (2013). Local atomic order in the melt and solid-liquid interface effect on the growth kinetics in a metallic alloy model. Phys. Rev. Lett..

[17] Kresse G, Furthmüller J (1996). Efficiency of ab-initio total energy calculations for metals and semiconductors using a plane-wave basis set. Comput. Mater. Sci..

[18] LAMMPS code, http://lammps.sandia.gov/, Plimpton, S. J. Fast parallel algorithms for short-range molecular dynamics. *J. Comp. Phys*. **117**, 1–19 (1995).

[19] Jakse N, Nguyen TLT, Pasturel A (2011). Ordering effects in disordered systems: the Au–Si system. J. Phys.: Condens. Matter.

[20] Jakse N, Nguyen TLT, Pasturel A (2012). Local order and dynamic properties of liquidAu_x_Si_1−x_ alloys by molecular dynamics simulations. J. Chem. Phys..

[21] Honeycutt JD, Andersen HC (1987). Molecular dynamics study of melting and freezing of small Lennard-Jones clusters. J. Phys. Chem..

[22] Daudin, R. Formation and Supercooling of AuSi eutectic droplets on Si substrates: an *in-situ* using synchrotron radiation. *PhD thesis, Univ. Grenoble* (2012).

[23] Anatharaman TR, Luo HL, Klement W (1966). Formation of new intermetallic phases in binary eutectic systems by drastic undercooling of the melt. Nature.

[24] Chen HS, Turnbull D (1967). Thermal properties of Gold-Silicon binary alloy near the eutectic composition. J. Appl. Phys..

[25] Andersen GA, Bestel JL, Johnson AA, Post B (1971). Eutectic deposition in the gold-silicon system. Mater. Sci. Eng..

