# Supplementary material for: Effect of pentagonal-coordinated surface on crystal nucleation of an undercooled melt
Source: Sci Rep. 2018 Sep 25;8:14314. doi: 10.1038/s41598-018-32594-w (PMC6156225; doi:10.1038/s41598-018-32594-w)
Supplement: Supplementary file 1 — Supplementary Information [file 41598_2018_32594_MOESM1_ESM.pdf]

Supplementary Information

Effect of pentagonal-coordinated surface on the crystal nucleation of an undercooled melt

A. Pasturel and N. Jakse

Univ. Grenoble Alpes, CNRS, Grenoble INP, SIMaP, F-38000 Grenoble, France

1. Simulation Methods

a. **Ab initio molecular dynamics simulations.** The *ab initio* molecular dynamics (AIMD) simulations were carried out using the DFT as implemented in the Vienna *ab initio* simulation package [1]. Projected augmented plane waves [2] (PAWs) with the Perdew-Wang exchange-correlation potentials have been adopted. The valence state of each element has been defined previously in the provided PAW potentials and the planewave cutoff is 245 eV. All the dynamical simulations were carried out in the canonical ensemble by means of a Nosé thermostat to control temperature. Newton's equations of motion were integrated using the Verlet algorithm in the velocity form with a time step of 1 fs. Only the Γ-point was considered to sample the supercell Brillouin zone. The simulations procedures are described below.

b. **Classical molecular dynamics simulations.** Classical molecular dynamics simulation were performed using interatomic interactions built within the Modified Embedded-Atom Model (MEAM) designed for the Au-Si system [3, 4]. The MEAM formalism is well documented in the literature therefore we refer the reader to Refs. [5-8] for a detailed description. Here we recall only the main feature for a comprehensive purpose. In the MEAM formalism, the potential energy functional can be expressed as

$$E = \sum_i \left\{ F_i(\bar{\rho}_i) + \frac{1}{2}\sum_{i \neq j} S_{ij}\Phi_{ij}(r_{ij}) \right\} \quad (1)$$

where $F_i(\bar{\rho}_i)$ is the embedding energy function depending on the background electronic density ρ_i. The function $\Phi_{ij}(r_{ij})$ represents the pair potential interaction as a function of interatomic distance r_{ij} between atoms *i* and *j*, and S_{ij} is a screening factor. Briefly, the electron density is calculated first for each atomic site from an analytic expression taking into account the length and directionality in bonding of the neighbors. Then, from a specific form of the embedding function and the total energy, estimated using the equation of state of Rose at 0 K for a given reference structure, the pair potential is determined as a function of the interatomic distance using Eq. (1).

For Au-Si alloys we consider the model of Ryu and Cai [9, 10] based on the fitting of the experimental Au-Si phase diagram and a refinement of existing MEAM potentials for the pure elements Au and Si. Here we use a further refinement of this potential done in [3, 4], leading to a good agreement with AIMD simulations.

With this MEAM potential we have performed molecular dynamics simulations using the LAMMPS code [11] to investigate the structural and dynamic properties of the of Si(001)/ AuSi and Si(111)-(6×6)/ AuSi interfaces as described below. As for the AIMD simulations, the classical molecular dynamics (MD) simulations were done using the Verlet algorithm in the velocity form with a time step of 1 fs. The simulation were conducted in the canonical ensemble (NVT ensemble) using a Nose thermostat.

2. Design of the solid/liquid interfaces and simulation procedures

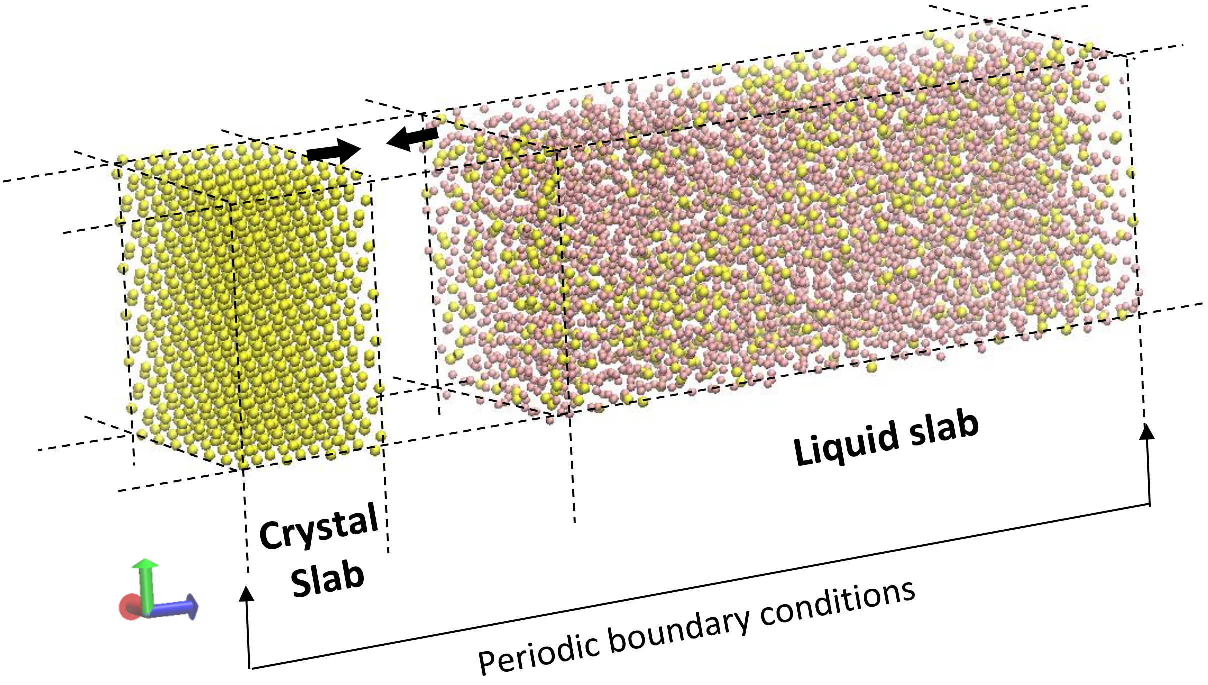

Figure S1: Schematic view of the interface design. The axis orientations of the simulation boxes are given by the red (x-axis), green (y-axis) and blue (z-axis) thick arrows. Si atoms are in yellow color, and Au atoms are in pink color.

The design of the solid/liquid interface is described schematically in Figure S1, which was done either by AIMD and classical MD. The liquid and crystalline part of

the system are simulated independently by molecular dynamics simulation à T = 700 K, with the same size in x and y directions so that equilibrium configurations can be directly assembled. The number of atoms of the eutectic Au-Si liquid was chosen to match the composition and experimental density [12]. An appropriate length in the z-direction is also chosen. The specific size of each simulation box is described below. A simulation length of 20 ps (AIMD and MD) were sufficient to equilibrate the liquid and the crystalline systems.

After equilibration, the liquid and crystalline parts were assembled, as shown in Figure S1, with an initial separation distance between the two parts chosen to correspond to the Au-Si distance found in the liquid alloy [3, 4]. Periodic boundary conditions were applied on the opposite faces, by fixing correctly the length of the simulation box in the z direction. Of course, periodic boundary conditions are also kept in the x and y directions. In doing so, the system is constituted by successive liquid and solid slabs in the z direction, and each liquid slab has two interfaces with the solid.

Note that for the Si(111)-(6×6) / AuSi interface, a (6×6) Au layer is deposited on the (x-y) plane on both sides of the (111) oriented Si crystal, and further relaxed by a conjugated gradient minimization technique before assembling to liquid slab.

For AIMD simulations the characteristics of the supercell for the liquid/solid interface simulations are the following:

- Si(001) / AuSi interface: parallelepiped supercell with size L_x=16.29 Å, L_y = 16.29 Å and L_z = 33.1 Å, and the number of atoms is 403 (220 Au and 183 Si).
- Si(111)-(6×6) / AuSi interface: triclinic supercell imposed by the (6×6) Au layer, with size L_x=23.18 Å, L_y = 20.07 Å and L_z = 38.5 Å, with a xy tilt of 11.52 Å. The number of atoms is 616 (349 Au and 267 Si).

For classical MD simulations the characteristics of the supercell for the liquid/solid interface simulations are the following:

- Si(001) / AuSi interface: parallelepiped supercell with size L_x=38.01 Å, L_y = 32.58 Å and L_z = 108.3 Å, and the number of atoms is 7000 (4097 Au and 2903 Si).

- Si(111)-(6×6) / AuSi interface: triclinic supercell imposed by the (6×6) Au layer with size L_x=46.08 Å, L_y = 39.91 Å and L_z = 117 Å, with a *xy* tilt of 23.02 Å. The number of atoms is 11088 (6943 Au and 4145 Si).

The solid/liquid interface is then equilibrated again during a few ps. For the simulations at T = 600 K and below, the equilibrated solid/liquid interface obtained at T = 700 K was cooled down with a cooling rate of 10^{12} K/s, by a linear temperature ramp. The total simulation time after equilibration for all temperature is 110 ps for AIMD runs and 30 ns for classical MD. At each temperature, the box size L_z of the solid/liquid interface is slightly reduced to maintain the experimental density in the liquid slab.

3. Calculation of the self-diffusion coefficients

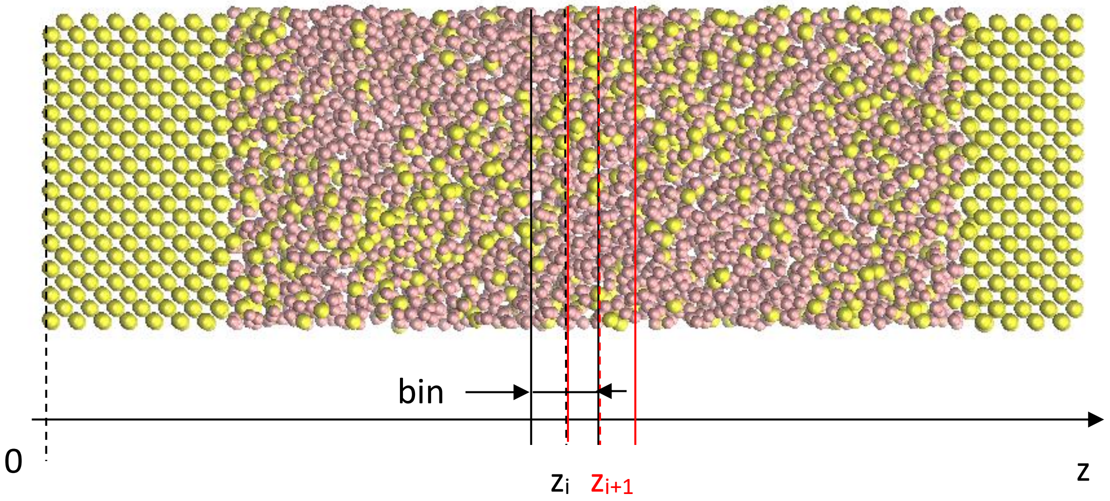

Figure S2: Schematic view of the interface. The axis orientations of the simulation boxes are given by the red (x-axis), green (y-axis) and blue (z-axis) thick arrows. Si atoms are in yellow color, and Au atoms are in pink color.

The self-diffusion coefficient is obtained by standard techniques [13] from the slope at long times of the mean-square displacement (MSD)

$$\left\langle R^2(t)\right\rangle = \frac{1}{N}\left\langle \sum_{i=1}^{N}\left[\mathbf{r}_i(t) - \mathbf{r}_i(t=0)\right]^2 \right\rangle, \quad (1)$$

where $\mathbf{r}_i(t)$ denotes the position of atom *i* at time *t*. Of interest here is the determination of the self-diffusion coefficients as a function of the distance from the interface to study its influence. Figure S2 shows a schematic representation of the solid liquid interface in which the liquid part is divided in bins of 10 Å width, placed each 5 Å (as can be seen there is an overlap of adjacent bins).

For each bin, an individual MSD $[\mathbf{r}_i(t) - \mathbf{r}_i(t=0)]^2$ is calculated for each atom *i* located in the z-range of the bin. The calculation of the individual MSD of a given atom is stopped as soon as the atom leaves the bin. For all the atoms in the bin whose individual MSD is meaningful (*i.e.* a linear slope of the individual MSD is achieved) an averaged MSD is calculated and self-diffusion $D(z_i)$ is determined for the distance z_i corresponding of the center of the bin. The calculation is repeated for independent time segments during the simulation and averaged to increase the statistical accuracy.

4. Layer structure of Si(001) / AuSi and Si(111)-(6×6) / AuSi interfaces.

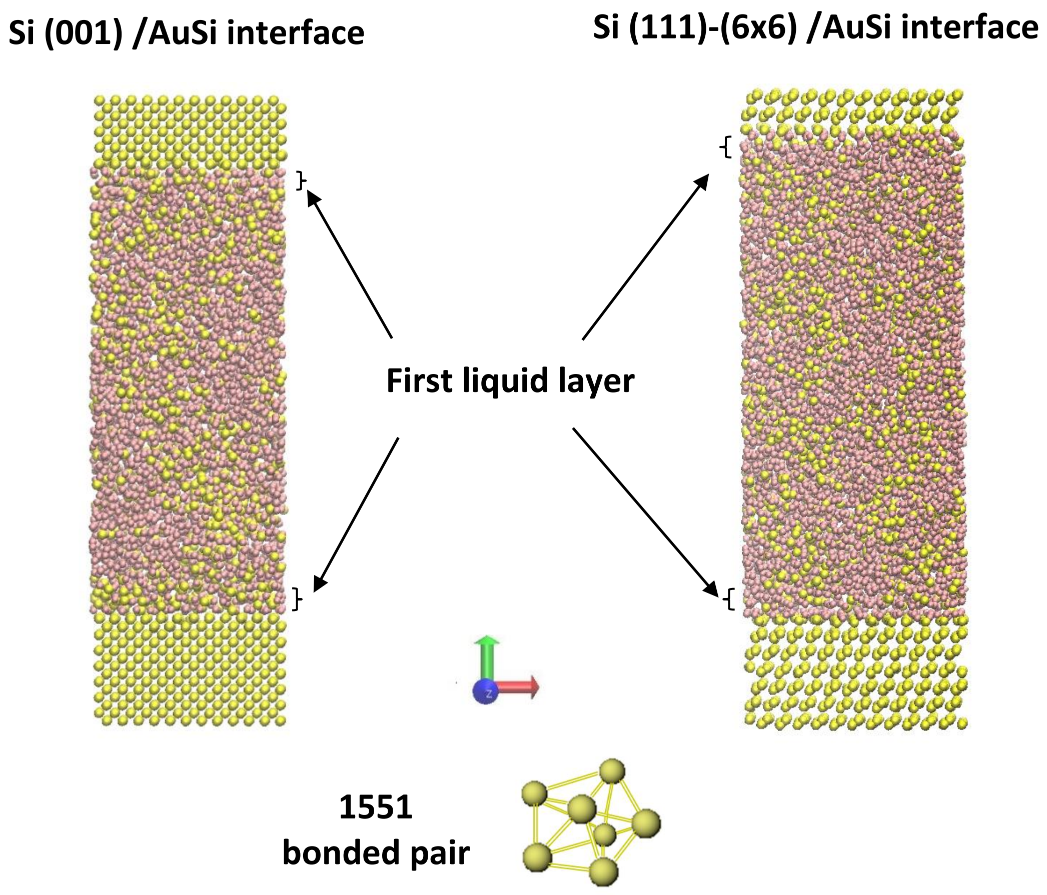

Figure S3: Snapshot of the simulation box (Si: yellow color, and Au: pink color). The axis orientations for both simulation boxes are given by the red (*x*-axis), green (y-axis) and blue (z-axis) thick arrows. A typical 1551 bonded pair found in the liquid slab is also shown.

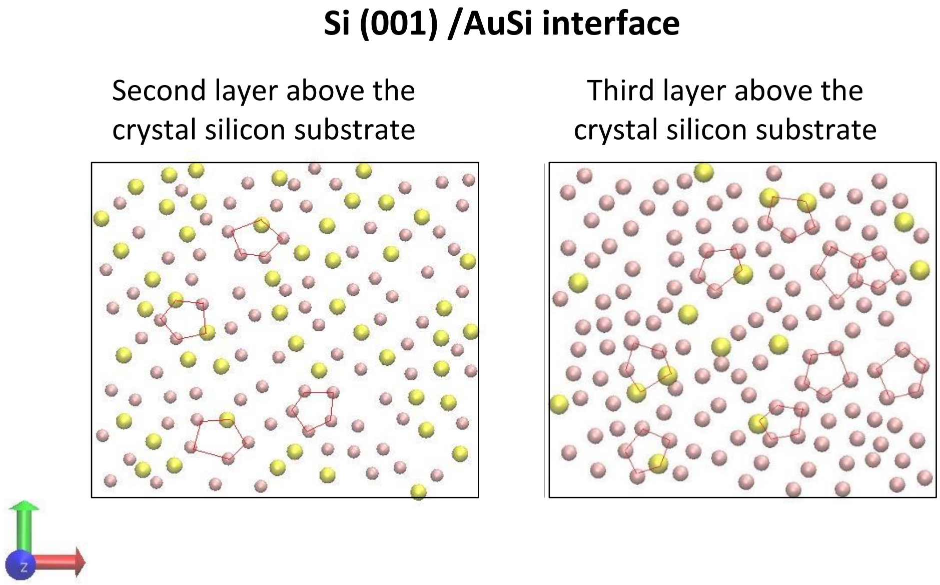

Figure S4: layer structure Si (001) /AuSi interface at *T* = 700 K. The axis orientations of the simulation boxes are given by the red (x-axis), green (y-axis) and blue (z-axis) thick arrows. Si atoms are in yellow color, and Au atoms are in pink color. pentagonal atomic configurations are highlighted with red lines.

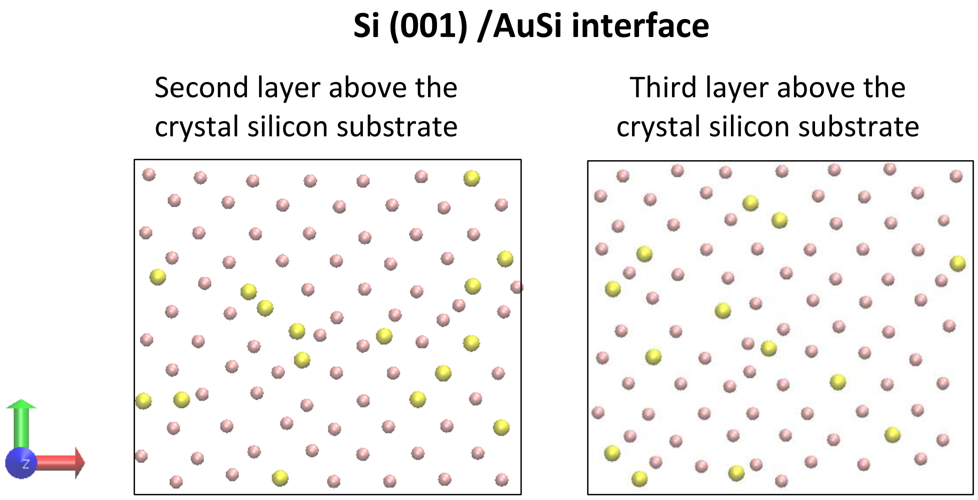

Figure S5: layer structure Si (001) /AuSi interface at *T* = 600 K. The axis orientations of the simulation boxes are given by the red (x-axis), green (y-axis) and blue (z-axis) thick arrows. Si atoms are in yellow color, and Au atoms are in pink color.

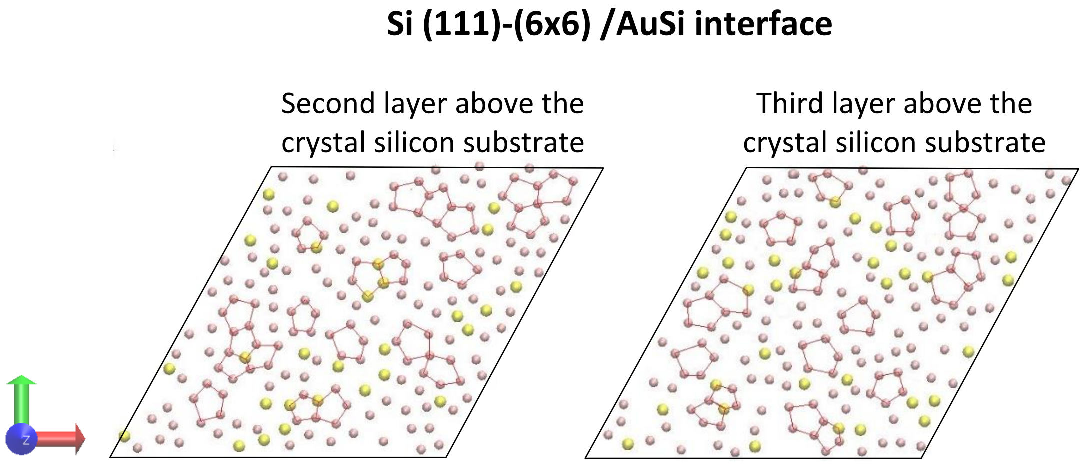

Figure S6: layer structure of the Si(111)-(6×6) / AuSi interfaces at T = 600 K. The axis orientations of the simulation boxes are given by the red (x-axis), green (y-axis) and blue (z-axis) thick arrows. Si atoms are in yellow color, and Au atoms are in pink color. pentagonal atomic configurations are highlighted with red lines.

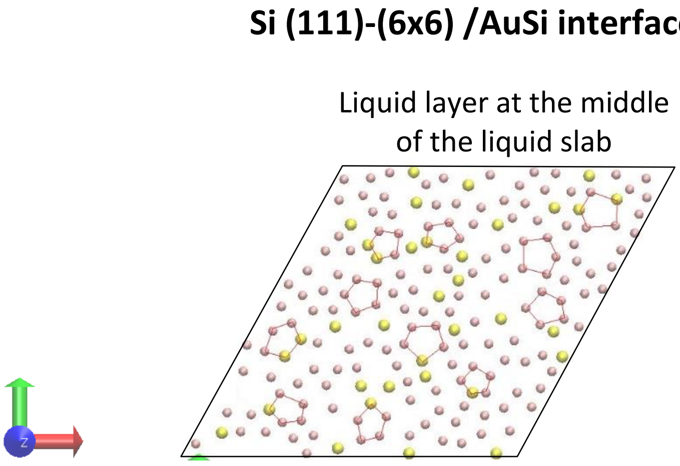

Figure S7: liquid layer structure of the Si(111)-(6×6) / AuSi interfaces at T = 600 K. The axis orientations of the simulation boxes are given by the red (x-axis), green (y-axis) and blue (z-axis) thick arrows. Si atoms are in yellow color, and Au atoms are in pink color. Pentagonal atomic configurations are highlighted with red lines.

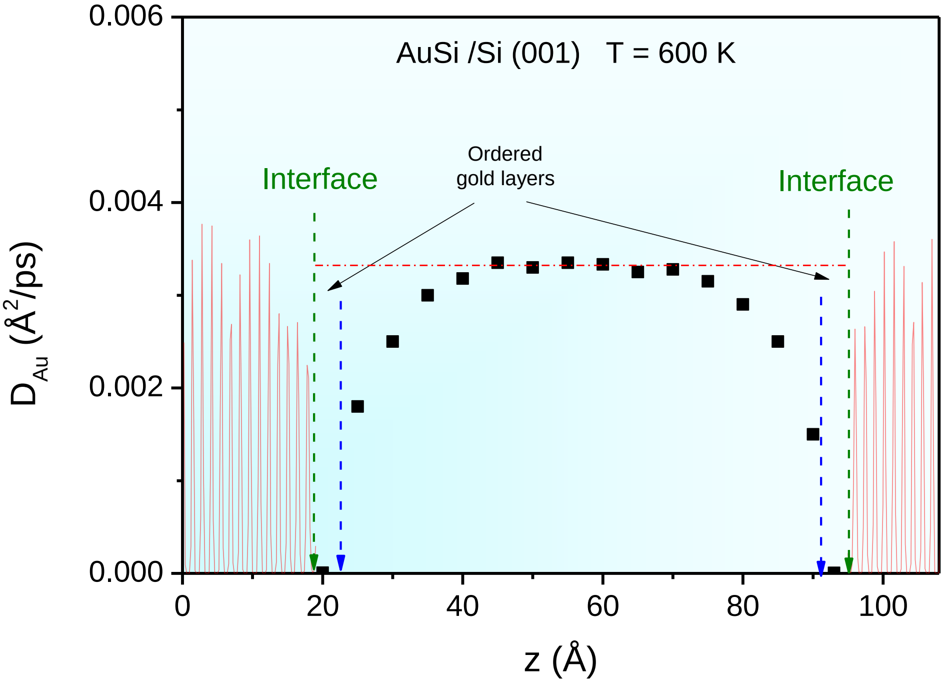

Figure S8: Self-diffusion coefficient for Au as a function of *z*, calculated in the liquid slab from the large scale classical MD simulations with the MEAM potential at T = 600 K for the Si (001) / AuSi interface. The shadowed red lines in both panels represent the density profiles in arbitrary units of the crystalline substrate for visual purpose only, in order to highlight the AuSi liquid slab *z*-range. The green dotted line arrows mark the positions of both interfaces, and the red dot-dashed horizontal line corresponds to the diffusion value of the bulk eutectic liquid [4].

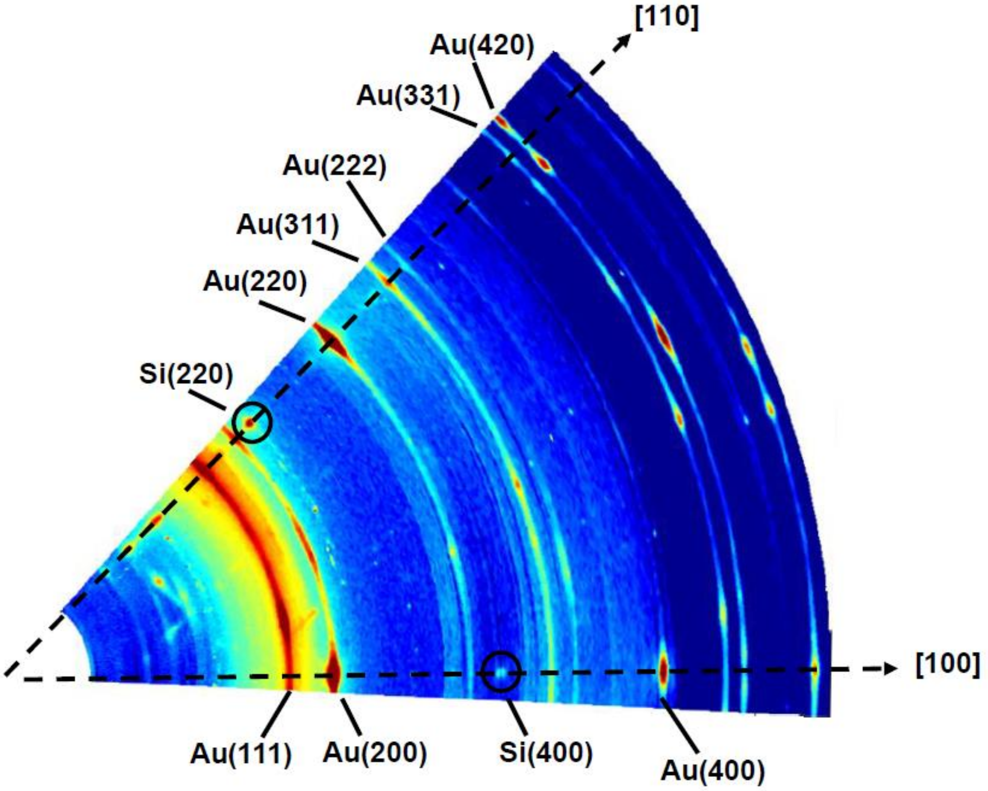

Figure S9: Reciprocal space map of solidified Au on Si(001) substrate from surface X-ray scattering, after Ref. 14 (p. 119) with permission of the author. It is seen that the solid gold displays a “cube on cube” epitaxy: the Au(220) ring intensity is concentrated on the Si[110] direction while the majority of the Au(200) intensity (as well as the of Au(400)) is located on the Si[100] azimuth. Therefore, the clearly preferred orientation relationship between the solidified Au and the Si(001) surface is : [100]Au(001)||[100]Si(001).

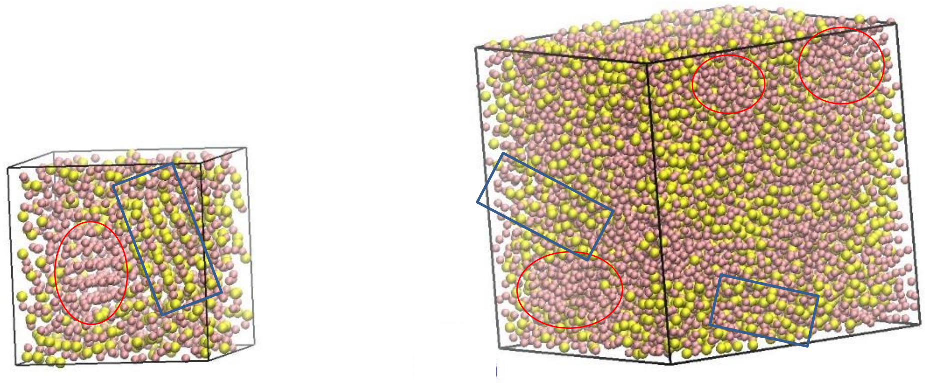

Figure S10: Homogeneous nucleation event from classical molecular dynamics simulations of liquid AuSi eutectic liquid at T = 590 K. The number of atoms in the cubic simulation cell is 1372 for the left panel and 10976 in the right panel. The system was quenched at a cooling rate of 10^{12} K/s from an equilibrium liquid at T = 700 K. The run was continued at T = 590 K for 12 ns and 15 ns, respectively, before nucleation occurs. Appearance of Au grains as well as Au_3Si metastable phase in both simulations show that there are no size effects in this phenomenon. Si atoms are in yellow color, and Au atoms are in pink color. The red circles highlight some of gold grains formed and blue rectangles show some grains of Au_3Si metastable phase.

References

[1] Kresse, G. & Furthmüller, J. Efficiency of ab-initio total energy calculations for metals and semiconductors using a plane-wave basis set. *Comput. Mater. Sci.* **6**, 15–50 (1996)

[2] Kresse, G. & Joubert, D. From ultrasoft pseudopotentials to the projector augmented-wave method. *Phys. Rev. B* **59**, 1758–1775 (1999)

[3] Jakse, N. Nguyen, T.L.T & Pasturel, A. Ordering effects in disordered systems: the Au–Si system. *J. Phys.: Condens. Matter* **23**, 404205 (2011)

[4] Jakse, N. Nguyen, T.L.T & Pasturel, A. Local order and dynamic properties of liquid Au_xSi_{1-x} alloys by molecular dynamics simulations. *J. Chem. Phys.* **117**, 204504 (2012)

[5] Baskes, M. I. Modified embedded-atom potentials for cubic materials and impurities. *Phys. Rev. B* 46, 2727–2742 (1992)

[6] Baskes, M. I. Determination of modified embedded atom method parameters for nickel. *Mat. Chem. Phys.* **50**, 152 (1997)

[7] Lee, B. J. & Baskes, M. I. Second nearest-neighbor modified embedded-atom-method potential. *Phys. Rev. B* **62**, 8564–8567 (2000)

[8] Lee, B. J. Baskes, M. I. Kim, H. & Cho, Y. K. Second nearest-neighbor modified embedded atom method potentials for bcc transition metals. *Phys. Rev. B* **64**, 184102 (2001)

[9] Ryu, S. & Cai, W. A gold–silicon potential fitted to the binary phase diagram. *J. Phys.: Condens. Matter* **22**, 055401 (2010)

[10] Ryu, S. Christopher, R. Weinberger, C. R. Baskes, M. I. & Cai, W. Improved modified embedded-atom method potentials for gold and silicon. *Model. Simul. Matter Sci. Eng.* **17**, 075008 (2009)

[11] LAMMPS code (http://lammps.sandia.gov/), Plimpton, S. J. Fast parallel algorithms for short-range molecular dynamics. *J. Comp. Phys.* **117**, 1–19 (1995)

[12] Bian X., Qin J., Qin X., Wu Y., Wang C., and Thompson M. Structural features of liquid metallic glass former. *Physics Lett. A* **359**, 718 (2006).

[13] Allen M.P. and Tildesley D.J., Computer simulation of liquids, Oxford Science Publication (1989); Smit B. and Frenkel D., Understanding Molecular simulations, Second Edition, Academic Press (2002).

[14] Daudin, R. Formation and Supercooling of AuSi eutectic droplets on Si substrates: an in-situ using synchrotron radiation. *PhD thesis*, Univ. Grenoble (2012)
